# Copenhagen comorbidity in HIV infection (COCOMO) study: a study protocol for a longitudinal, non-interventional assessment of non-AIDS comorbidity in HIV infection in Denmark

**DOI:** 10.1186/s12879-016-2026-9

**Published:** 2016-11-26

**Authors:** Andreas Ronit, Judith Haissman, Ditte Marie Kirkegaard-Klitbo, Thomas Skårup Kristensen, Anne-Mette Lebech, Thomas Benfield, Jan Gerstoft, Henrik Ullum, Lars Køber, Andreas Kjær, Klaus Kofoed, Jørgen Vestbo, Børge Nordestgaard, Jens Lundgren, Susanne Dam Nielsen

**Affiliations:** 1 Viro-immunology Research Unit, Department of Infectious Diseases 8632, Rigshospitalet, University of Copenhagen, Copenhagen, Denmark; 2 Department of Infectious Diseases, Hvidovre Hospital, University of Copenhagen, Copenhagen, Denmark; 3 Department of Radiology, Rigshospitalet, University of Copenhagen, Copenhagen, Denmark; 4 Department of Clinical Immunology 2034, Blood Bank, Rigshospitalet, University of Copenhagen, Copenhagen, Denmark; 5 Department of Cardiology, The Heart Centre, Rigshospitalet, University of Copenhagen, Copenhagen, Denmark; 6 Department of clinical physiology, Nuclear Medicine and PET, Rigshospitalet, Copenhagen, Denmark; 7 Division of Infection, Immunity and Respiratory Medicine, University Hospital South Manchester NHS Foundation Trust and The University of Manchester, Manchester, UK; 8 Department of Clinical Biochemistry, Herlev and Gentofte Hospital, University of Copenhagen, Herlev, Denmark; 9 Faculty of Health and Medical Sciences, University of Copenhagen, Copenhagen, Denmark; 10 CHIP, Department of Infectious Diseases, Section 2100, Rigshospitalet, University of Copenhagen, Copenhagen, Denmark

**Keywords:** HIV, Comorbidity, Cardiovascular diseases, Respiratory disease, Liver related diseases, Inflammation

## Abstract

**Background:**

Modern combination antiretroviral therapy (cART) has improved survival for people living with HIV (PLWHIV). Non-AIDS comorbidities have replaced opportunistic infections as leading causes of mortality and morbidity, and are becoming a key health concern as this population continues to age. The aim of this study is to estimate the prevalence and incidence of non-AIDS comorbidity among PLWHIV in Denmark in the cART era and to determine risk factors contributing to the pathogenesis. The study primarily targets cardiovascular, respiratory, and hepatic non-AIDS comorbidity.

**Methods/design:**

The Copenhagen comorbidity in HIV-infection (COCOMO) study is an observational, longitudinal cohort study. The study was initiated in 2015 and recruitment is ongoing with the aim of including 1500 PLWHIV from the Copenhagen area. Follow-up examinations after 2 and 10 years are planned. Uninfected controls are derived from the Copenhagen General Population Study (CGPS), a cohort study including 100,000 uninfected participants from the same geographical region. Physiological and biological measures including blood pressure, ankle-brachial index, electrocardiogram, spirometry, exhaled nitric oxide, transient elastography of the liver, computed tomography (CT) angiography of the heart, unenhanced CT of the chest and upper abdomen, and a number of routine biochemical analysis are uniformly collected in participants from the COCOMO study and the CGPS. Plasma, serum, buffy coat, peripheral blood mononuclear cells (PBMC), urine, and stool samples are collected in a biobank for future studies. Data will be updated through periodical linking to national databases.

**Discussion:**

As life expectancy for PLWHIV improves, it is essential to study long-term impact of HIV and cART. We anticipate that findings from this cohort study will increase knowledge on non-AIDS comorbidity in PLWHIV and identify targets for future interventional trials. Recognizing the demographic, clinical and pathophysiological characteristics of comorbidity in PLWHIV may help inform development of new guidelines and enable us to move forward to a more personalized HIV care.

**Trial registration:**

ClinicalTrials.gov: NCT02382822.

**Electronic supplementary material:**

The online version of this article (doi:10.1186/s12879-016-2026-9) contains supplementary material, which is available to authorized users.

## Background

The global scale-up of combination antiretroviral therapy (cART) has contributed to a 35% decline in acquired immune deficiency syndrome (AIDS)-related deaths since 2005 [1], and averted almost 8 million deaths worldwide [2]. As a consequence, the median age of people living with HIV (PLWHIV) has steadily increased and is modelled to be almost 60 years in 2030 [3]. The impact of cART on non-AIDS defining conditions has been less impressive, and prevalence of non-AIDS comorbidity in PLWHIV is high [3–5]. Multiple factors may explain the increased burden of non-AIDS comorbidities, including overrepresentation of traditional risk factors [6], and chronic immune activation and inflammation [7]. Toxicities associated with the long-term exposure to cART may also be associated with non-AIDS comorbidity [8], although early initiation of cART may lower the risk of non-AIDS related events [9].

The changing disease spectrum will have implications for clinical HIV care, a prospect that will put new demands on the health care system, and warrants further studies to assess the burden of disease and risk factors involved. Primary outcome measures in the Copenhagen Comorbidity in HIV-infection (COCOMO) study are characterized with respect to three biological systems, i.e. cardiovascular, respiratory, and hepatic non-AIDS comorbidity.

Cardiovascular disease (CVD) has become one of the leading causes of mortality in PLWHIV [10, 11]. HIV may be an independent risk factor even when controlling for traditional CVD risk factors [12, 13]. Moreover, CVD-related proportionate mortality may be rising [14]. Large cohort studies including uninfected individuals and multiple CVD endpoints are warranted to ascertain further insight into the risk factors and mechanisms driving CVD in PLWHIV in the current cART era.

Respiratory non-AIDS comorbidity in PLWHIV has received less attention. Smaller clinic-based cross-sectional studies using pulmonary function testing have been conducted [15, 16]. Few studies have also assessed pulmonary function longitudinally [17–20]. Data from these studies indicate an increased risk of non-AIDS respiratory comorbidity in PLWHIV. Thus, PLWHIV may be more susceptible to the harms of smoking than the general population, but the mechanistic factors behind the proposed increased risk of respiratory comorbidity have not been thoroughly investigated.

Liver related deaths in PLWHIV are often related to hepatitis C virus (HCV) or hepatitis B virus (HBV), but may also occur in the absence of chronic viral hepatitis [21]. This may be due to the presence of multiple risk factors including high alcohol consumption, metabolic disorders driven by HIV, viral replication, and use of cART [22–25]. Studies have also shown that non-alcoholic fatty liver disease (NAFLD) and fibrosis is associated with duration of HIV, persistent HIV viral replication, cART use, as well as genetic variants [26–29]. As with CVD and respiratory comorbidity, these results suggest contributory effects of HIV-related factors.

## Study objectives

The overall objective of the COCOMO study is to assess the burden of non-AIDS comorbidity in PLWHIV, and to gain further insight into the aetiology of non-AIDS comorbidity. We wish to identify both HIV-related and HIV un-related risk factors involved in the pathogenesis of non-AIDS comorbidity.

- AIM 1: To determine if prevalence and incidence of cardiovascular, respiratory, and hepatic diseases in HIV-infected persons are different from what is found in demographically similar uninfected persons when both groups have uniformly measured clinical data and information regarding risk factors. Clinically measured primary outcomes include 1) non-calcified plaques detected by cardiac CT angiography, 2) dynamic lung function indices measured by spirometry, and 3) liver fibrosis measured by transient elastography (TE). Several other outcome measures are included (Table 1).

**Table 1:** Clinical measurements and collection of biomaterial

Measurements	Description
Anthropometry	Height: Stadiometer (Soehnle, Nassau, Germany) without shoes^a^ Weight: Scale (Soehnle, Nassau, Germany) without shoes or heavy clothing^a^ Waist and hip circumference: Measuring tape (SECA, Birmingham, UK)^a^
Ankle-brachial index	Doppler (Sonotrax Basic A, Edan, San Diego, CA, U.S.)^a^
Blood pressure	IntelliVue MP5SC (Philips, Amsterdam, Netherlands)^a^
Electrocardiogram	Cardiosoft V6.73 (GE Healthcare, Buckinghamshire, UK)^a^
Spirometry	EasyOne® ultrasonic spirometer (ndd Medical, Zürich, Switzerland)^a^
eNO	NIOX VERO® (Aerocrine AB, Solna, Sweden)^c^
Transient elastography	Fibroscan (Fibroscan 402, Echosens^TM^, Paris, France)^a^
CT scans	CT angiography, CACS, CT chest, CT upper abdomen (Aquillion One scanner, Toshiba Medical Systems, Otawara-shi, Tochigi-ken, Japan)^a^
Blood (non-fasting)	Peripheral venous blood: Buffy coat^a^, PBMC^b^, plasma^a^, serum^a^
Urine (spot, non-fasting)	Collected in sterile polypropylene tube (Sarstedt, Nürnbrecht, Germany)^c^
Stool (non-fasting)	Collected in polypropylene tube (Sarstedt, Nürnbrecht, Germany)^c^

^a^Collected uniformly in the Copenhagen Co-morbidity in HIV infection (COCOMO) Study cohort and in the Copenhagen general population study (CGPS). ^b^Collected in the COCOMO study cohort only. ^c^Collected uniformly in a subsample of the COCOMO cohort and in the CGPS, see text

*Abbreviations*: *CACS* coronary artery calcium score, *CT* computed tomography, *eNO* exhaled nitric oxide and *PBMC* peripheral blood mononuclear cells. See Methods for further details
- AIM 2: To identify HIV-related and HIV-unrelated risk factors that contribute to the pathogenesis of cardiovascular, respiratory, and hepatic diseases in HIV-infected persons and to study the interaction between these.

Hypotheses are registered at clinicaltrials.gov (NCT02382822).

## Methods/design

### Study design

The COCOMO study is a non-interventional, observational, longitudinal study initiated in March 2015. The study aims to enroll up to 1500 PLWHIV from two sites in Copenhagen (Rigshospitalet, University of Copenhagen, and Hvidovre Hospital, University of Copenhagen). Uninfected controls will be derived from the Copenhagen General Population Study (CGPS) with a study population of more than 100,000 participants from the greater Copenhagen area [30, 31]. An overview of physical measurement and collection of biomaterial (Table 1), hematological and biochemical measures (Table 2), and content of questionnaire are given (Table 3).

**Table 2:** Hematological and biochemical measures

Measurements	Description
Hematology	Hemoglobin, hematocrit, leucocytes, differential count, thrombocytes, red cell dimensions
Electrolytes and iron metabolism	Sodium, potassium, calcium, iron, magnesium, ferritin, transferrin
Liver/ organ related	Aspartate aminotransferase, alanine aminotransferase, alkaline phosphatase, bilirubin, gamma glutamyltransferase, albumin, antitrypsin, amylase, creatinine kinase, lactate dehydrogenase
Coagulation	Activated partial thromboplastin time, coagulation factor II+ VII+ X, D-dimer, fibrinogen
Lipids	Total cholesterol, remnant cholesterol, HDL-cholesterol, LDL-cholesterol, triglycerides, adiponectin, lipoprotein, apolipoprotein A, B, and E
Metabolic	Glucose, HbA1c, ethanol
Renal function	Creatinine, urea, uric acid
Thyroid function	Thyroid stimulating hormone, free T3/T4
Immunology	Immunoglobulin A and E, rheumatoid factor IgM, complement C3
Inflammation	High sensitivity C-reactive protein

All measures are analyzed uniformly in the Copenhagen co-morbidity (COCOMO) study cohort and in the Copenhagen general population study (CGPS) at Biochemical Department, University Hospital Herlev, Copenhagen

**Table 3:** Overview of variables recorded by questionnaires

Variable	Examples
External risk factors	
Smoking	Current, previous, and passive smoking, cumulative pack-years, type, filters, e-cigarettes
Alcohol/drug abuse	Current and previous alcohol intake, intravenous drug use, cannabis, methadone treatment
Biomass fuel/environmental exposure	Occupational dusts, organic, inorganic gasses, vapors, and fumes (outdoor and indoor)
Demographics	Ethnicity, nationality, marital status, children
Education/income	Level of education, house-hold income
Work	Employment, income, night work
Diet	Meat intake, vegetable intake
Daily living	Physical activity, social support, sleeping, stress, sun exposure
Medication use	Prescription and doses
Internal risk factors	
Gender and ethnicity	Ethnicity, place of birth
Perinatal/childhood events	Birthweight, delivery at term, breast feeding, respiratory tract infections
Family history of disease	AMI, apoplexy, asthma, COPD, hypertension, diabetes, cancer, depression
Questions for females	Pregnancies, menarche- and menopause age, abortions
Self-reported disease	Asthma, COPD, allergy, diabetes
Symptoms	Angina, dyspnea (MRC), cough, wheeze, sputum, reflux

Risk factors for non-AIDS comorbidity, self-reported disease, and subjective morbidity are assessed. Under each variable a non-exhaustive list of examples are given

*Abbreviations*: *AMI* acute myocardial infarction, *COPD* chronic obstructive pulmonary disease and *MRC* medical research council dyspnea scale

### Follow up examinations

**Fig. 1:**
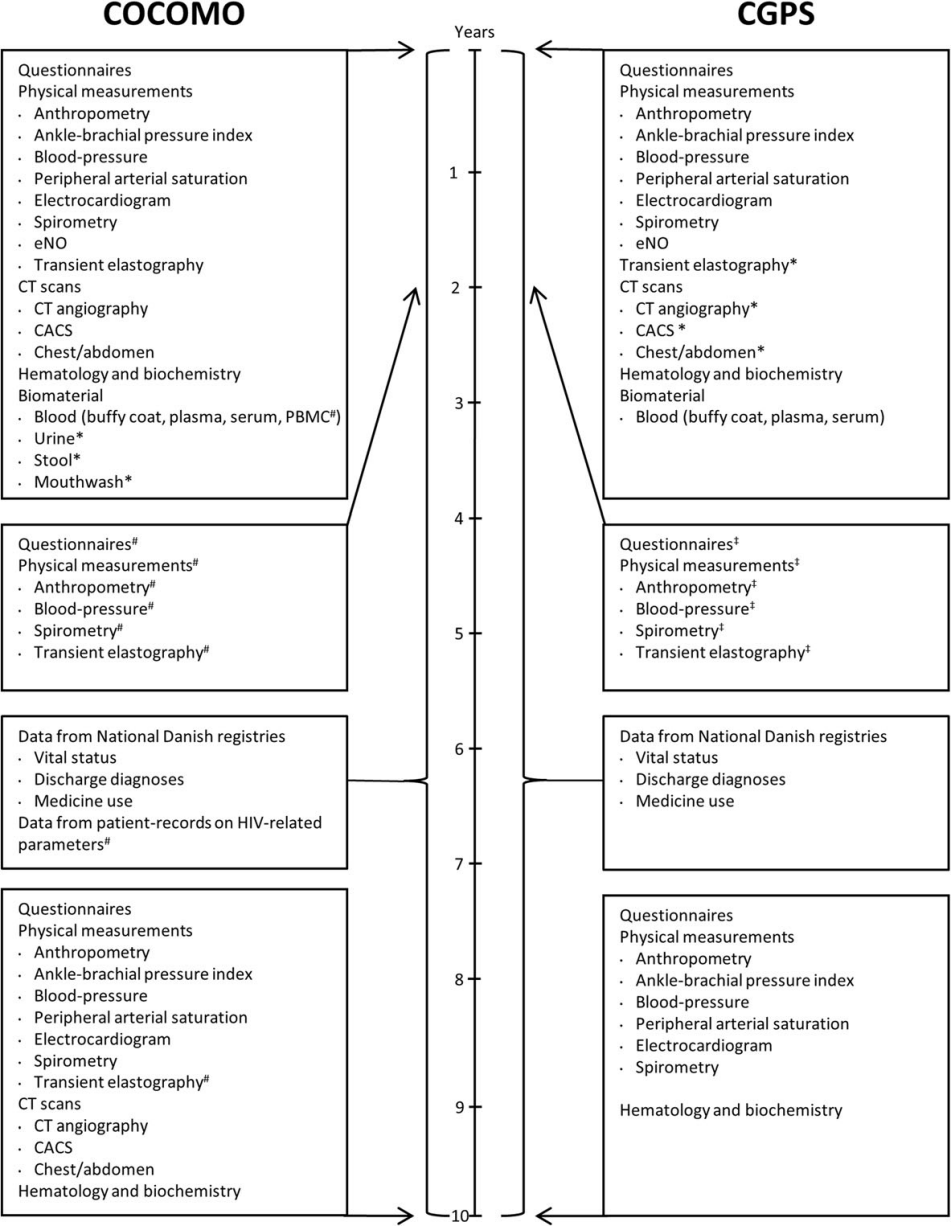
A timeline of the data collection in the Copenhagen Comorbidity in HIV (COCOMO) Study and the Copenhagen General Population Study (GCPS). ^#^ Collected in the COCOMO study cohort only. *Collected in a subsample of the cohort. ^‡^ Collected in a subsample of the CGPS cohort. Abbreviations: CACS (coronary artery calcium score), CT (computed tomography), eNO (exhaled nitric oxide), and PBMC (peripheral blood mononuclear cells). See Methods for further details

Each participant in the COCOMO study and CGPS will be evaluated at baseline and repeat evaluations will be performed after 10 years for all clinically measured outcomes, except CT scans in the CGPS. For a detailed description of follow up analysis see Fig. 1. In addition, follow up examinations in the COCOMO study, and in a subsample of the CGPS (*n* = 500), will be performed after 2 years and include pulmonary function testing and transient elastography (TE).

### Participants

#### Eligibility criteria

To be eligible for the COCOMO, study participants must be a minimum age of 18 years, infected with HIV-1, out-patient at either Department of Infectious Diseases at University Hospital Copenhagen or Hvidovre University Hospital, and be able to sufficiently understand oral and written study information in Danish or English to provide an informed consent. Insufficient knowledge of the Danish language does not exclude individuals if questionnaires can be filled out by the help of relatives or staff. Contraindications to the various measures performed in the study (i.e. renal impairment or contraindications to beta blocking agents used for CT angiography) do not exclude a participant from other parts of the study. In total, 4500 PLWHIV are eligible for inclusion. This number comprises ~95% of PLWHIV in Eastern Denmark/Zealand (inhabited by approximately 2,5 mill people). To be eligible for participation in the CGPS, individuals must be a minimum of 20 years of age and reside in the greater Copenhagen area. Starting from 2014 a large number of the first 100,000 participants from the CGPS were re-invited by regular mail. Of all inhabitants in the selected area, 25% of the 20–40 years old and 100% of the >40 years old are invited. A random sample of approximately 10,000 individuals above 40 years of age will have an unenhanced coronary artery calcium score (CACS) and CT angiography of the heart, and approximately 2000 will, in addition, have a CT scan of the chest and upper abdomen as described under methods. Scanning protocols in the COCOMO and CGPS studies are identical.

#### Sample size

The study includes several end-points, and the necessary sample size varies accordingly. One of the calculations used to estimate the required sample-size is based on forced expiratory volume in one-second (FEV_1_) decline, because there is existing solid longitudinal data on this endpoint, and because there is a natural age associated decline of FEV_1_. Sample size was estimated aiming to find a difference in the rate of forced expiratory volume in one-second (FEV_1_) decline per year between PLWHIV vs. uninfected controls. The standard deviation (SD) of FEV_1_ decline was derived from large general population studies and estimated to be SD = 60 mL/year [32, 33]. A difference (Δ) of 8 mL/year in HIV-infected vs. uninfected, allocation ratio of 1:4 (HIV-infected: uninfected), power = 0.9, and alpha level = 0.05, would require approximately 750 PLWHIV and 3000 controls. A clinically more relevant decline would require a smaller sample, e.g. Δ: 10 mL/year would require approximately 500 PLWHIV under the same conditions. A power graph for various Δs and powers can be found in Additional file 1 along with power calculations for other outcomes. Estimations were conducted using G*Power 3.1 and R 3.2.0 [34, 35].

### Planned statistical analysis

Prevalence and risk factors for non-AIDS comorbidity will be calculated based on baseline data. For some outcome data we plan to match PLWHIV with uninfected control 1:5 on age and gender. Student’s *t*-test, Mann-Whitney *U*-test and Pearson’s chi-squared test and appropriate uni- and multivariate regression analyses will be used at baseline. For prospective studies, Cox regression, Kaplan-Meier curves, log-rank test, and mixed effects models will be used. In general, missing data is expected to be low and multiple imputations not likely to be required. Characteristics for loss-to follow up will be assessed.

### Ethical considerations

All participants provide oral and written informed consent before study inclusion. The COCOMO study (H-8-2014-0004) and CGPS study (H-KF-01-144/01) have obtained approval from the Ethics Committee of the Capital Region and from the Danish Data Protection Agency. Although the study is not intended to be a screening project, participants are subjected to physical exams, laboratory tests, and imaging procedures. Screening is an area of controversy, may carry risks, and introduce false-positive and false-negative test results. Thus, each participant can choose to give consent to be contacted upon encounter of abnormal test results (e.g. anaemia, hypertension, renal impairment, pulmonary nodules, liver fibrosis). In such cases, participants will be referred for relevant follow up. All participants give a specific consent to storing of biological samples for future studies. The CT scan exposes participants to a radiation dose of five to a maximum of eight millisieverts (mSv). Although low, this radiation dose may cause an increase in lifetime risk of radiation-induced cancer. Before giving consent to the CT scan, participants have received both written and oral information about the radiation exposure and cancer risk. All adverse events and abnormal test results in the study are recorded.

### Physical measurements and collection of biomaterial in the COCOMO and CGPS study

The COCOMO and CGPS study collects all data uniformly. Blood pressure is measured twice electronically on right and left arm, respectively, with the participant in a relaxed seated position using IntelliVue MP5SC (Philips, Amsterdam, Netherlands). Electrocardiogram (ECG) is measured with a 12-lead ECG (Cardiosoft V6.73 GE Healthcare, Buckinghamshire, UK). Ankle-brachial-index (ABI) is measured in supine position in both extremities using a Doppler meter (Sonotrax Basic A 294534, Edan, San Diego, CA, US) by determining the systolic pressure of posterior tibial artery. Alternatively, the pressure of dorsalis pedis is determined. Pulse and peripheral arterial saturation is measured by fingertip puls-oximetry using IntelliVue MP5SC (Philips).

Spirometry is performed using the EasyOne® ultrasonic spirometer (ndd Medical, Zürich, Switzerland) with ndd spirettes (ndd Medical) in accordance with American Thoracic Society/European Respiratory Society guidelines [36], except that participants are standing in upright position without the use of a nose clip. The spirometer has an inbuilt calibration function that is checked regularly using a standardized 3-L syringe to confirm volume measurement to within ± 3% accuracy. In participants with a ratio between FEV_1_ and forced vital capacity (FEV_1_/FVC) less than 0.7, a repeat spirometry is conducted 15 min after bronchodilation with 400 μg salbutamol given by inhalation (Ventoline® Diskus, Glaxo Smith Kline, Middlesex, UK). In a subset of COCOMO participants (*N* = 425) and in the CGPS nitric oxide is measured in exhaled breath (eNO) using NIOX VERO® (Aerocrine AB, Solna, Sweden) [37].

Transient elastography (TE) (Fibroscan 402, Echosens™, Paris, France) is used to quantify the liver stiffness (LS) with the participant in supine position. A valid TE examination must meet the following criteria: At least 10 valid measurements, a success rate above 60% and an interquartile range (IQR) less than 30% of the median LS [38]. The scanner is calibrated regularly by the manufacturer.

### Collection of biomaterial in the COCOMO study only

Venous blood is collected non-fasting. The following blood components are collected and stored a) whole blood (ethylenediamine tetraacetic acid [EDTA]-anti-coagulated), b) buffycoat (EDTA-anti-coagulated), c) plasma (EDTA-anti-coagulated), and d) serum (Z Serum Sep Clot Activator). Venous blood for plasma samples is stored on ice until centrifugation in a cold-centrifuge at 4 °C. Buffycoats are transferred to 1 ml MAT-3741 2D screw tubes (Fisher Scientific, Hampton, NH, U.S.) and for RNA-tubes 10% DMSO is added.

Peripheral blood mononuclear cells (PBMCs) from all COCOMO participants are isolated (lithium-heparinized anti-coagulated), and cryopreserved within one hour of venipuncture. Leucosep® (Greiner Bio-One, Kremsmuenster, Austria) is used as separation media [39], and a freezing media of RPMI-1640, fetal bovine serum (FBS), and 10% DMSO [40] is used. Cells are frozen using a stepwise temperature decrease to −80 °C using CoolCell® (BioCision, Larkspur, CA, U.S.) [39]. Cryovials are transferred to liquid nitrogen within 72 h. A detailed protocol can be found elsewhere [41]. Approximately half of the COCOMO cohort is invited to provide a urine and/or stool sample. Non-fasting spot-urine samples are collected in a sterile polypropylene tube (Sarstedt, 60.549.001, Nürnbrecht, Germany) and stored at −80 °C within 4 h of sampling. Stool is collected by participants in a polypropylene tube with DNA stabilizer reagent (Stratec Molecular Gmbh, 1038111200, Berlin, Germany) and stored at −80 °C within three days of collection. In a smaller subset (*N* = 160) mouth wash has been collected by gargling of 10 mL saline (9 mg/mL) for one minute and stored at −80 °C.

### Computed tomography (CT) scans

The complete CT scan protocol includes a number of scans performed in the following sequence: Unenhanced CACS, scan of the upper abdomen, abdominal single slice acquisition for measurements of visceral adipose tissue, and low-dose chest CT. Finally, a contrast enhanced coronary angiography is performed with iodixanol (Visipaque®, GE Healthcare, Brøndby Denmark). If resting pulse exceeds 55 beats per minute (bpm), participants will be prepared for the coronary angiography with an orally administered β_1_-receptor blocker (Metocar, STADA Nordic, Herlev, Denmark). A maximum dose of 150 mg based on systolic blood pressure, weight, and pulse may be administered. Participants with moderate to severe COPD/asthma will receive Ivabradin 15 mg (Procoralan, Servier, Frederiksberg, Denmark) instead of a β1-receptor blocker. Only participants with a pulse below 70 bpm receive CT-angiography with intravenous contrast. Immediately before CT angiography is performed, one dose of 0.4 mg oral spray nitroglycerin (Nitrolingual, Pohl Boskamp, Hohenlockstedt, Germany) is administered. Contraindications to nitroglycerin include measured blood pressure less than 110 mmHg and concomitant treatment with other phosphodiesterase inhibitors. The maximum allowed radiation dose for the complete sequence is 8 mSv. Chest CT will not be performed in obese participants, where the expected radiation dose is higher than 8 mSv. All scans are performed on an Aquillion One scanner (Toshiba Medical Systems, Otawara-shi, Tochigi-ken, Japan). Scan parameters are described in Additional file 2.

### Questionnaires

All study participants complete a general health related paper questionnaire in Danish comprising more than one hundred items and a specific respiratory questionnaire. In the COCOMO study a specific questionnaire regarding current medication and illicit drug use is used. All items comprise questions on internal and external risk factors for development of non-AIDS comorbidities and information on self-reported symptoms and diseases. An overview of the variables used in the questionnaires is given in Table 3. Staff reviews completed questionnaires with each study participant for validation purpose. Questionnaires from the COCOMO and CGPS studies are scanned to automatically retrieve data.

### Other data sources

For the COCOMO study HIV-related variables will be retrieved from patient records. Information on vital status, medicine use and discharge diagnoses will be retrieved from the National Danish registries. The Civil Registration System (CRS) provides each resident of Denmark with a unique personal identifier and is updated daily with changes in vital status including date of emigration and date of death [42]. The Danish National Patient Register (NPR) contains information on all admissions to Danish Hospitals including discharge diagnosis according to the International Classification of Disease (ICD)–10 from 1994 [43]. The Registry of Medicinal Product Statistics contains all information on medicines since 1994, including all prescription and non-prescription drug sales from pharmacies and shops, and all deliveries from the general practitioners and hospitals [44].

## Discussion

Non-AIDS comorbidities are frequently encountered in the routine care of PLWHIV. As the number of PLWHIV has increased, and this population continues to age, information on the burden of comorbidity and potential risk factors leading to disease is warranted. The current study is an observational and longitudinal cohort study. It includes several clinical measures and is conducted in parallel to a large control cohort (CGPS) from the same geographical area using uniformly collected data. The primary goal is to identify the burden and the determinants of non-AIDS comorbidity. Primary outcome measures are characterized with respect to three biological systems, i.e. cardiovascular, respiratory, and hepatic non-AIDS comorbidity. Beyond the primary research questions, this study will establish a biobank for future prospective studies to assess pathogenesis of comorbidity in HIV infection.

Despite numerous studies on non-AIDS comorbidity, many questions remain: What is the burden of disease in the current cART era and which mechanisms are at play? What is the optimal clinical care, screening, and monitoring of patients with comorbidities? How does socioeconomic status affect development and prognosis on comorbidity? How will cART be tolerated in the elderly and what are the effects of polypharmacy in the elderly? In 2030, 28% of PLWHIV are expected to have three comorbidities or more, and 54% will be prescribed co-medication [3]. Most randomized trials of cART have, however, excluded elderly PLWHIV or those with comorbidities, and there is a continuous need to track non-AIDS comorbidity in non-interventional studies.

This study has *a priori* been designed to assess non-AIDS comorbidities. Thus, one of the main strengths is the multimodal assessment of risk factors associated with non-AIDS comorbidity. This will also be one of the largest cohort studies with assessment of non-AIDS comorbidity using clinical measured endpoints. Other cohort studies that are ongoing with use of various clinical examinations, include the Multicenter AIDS cohort study (MACS) [45], Women’s interagency HIV study (WIHS) [46], the AIDS linked to intravenous experience (ALIVE) study [47], all from the United States, the cohort from a HIV metabolic clinic in Italy [4], and most recently the AGE_H_IV cohort study from the Netherlands [48]. Besides the use of a control cohort with uniformly collected data, a main strength also includes the use of Danish registry data. These registries will allow linkage of data from the COCOMO study cohort to complete information on cause and date of death, hospital discharge, and of prescription medicine.

A weakness includes selection bias such as healthy volunteer bias, as those with serious illness or disability may be less likely to participate. Non-participation may include individuals that have difficulty attending on-site examinations (e.g. IDUs, elderly, home- or nursing home-bound). CGPS participants are recruited at random whereas COCOMO participants are recruited at their regular ambulatory visits. Exact reasons for non-participation will not be registered. However, the number of non-participaters will be registered, and to some extent, it will be possible to compare the non-participators with the participators, e.g. in respect to age, gender, CD4 count, and viral load. All PLWHIV registered as out-patients at the two sites receive an invitation to participate. Advertisement is found at the Patient Organization HIV Denmark (www.hiv-danmark.dk) and as posters at the two sites.

As the study collects information on some early life events through self-reports (i.e. exposure to passive smoking, childhood respiratory infections), a recall-bias cannot be precluded. However, few non-AIDS comorbidity studies have previously collected such information. Although the study is large, a sample size of 1500 PLWH precludes the study from examining rare clinical diagnoses such as specific non-AIDS cancers. A loss to follow up is anticipated for the clinically measured endpoints, and has been taken into account in power analysis. Finally, the study is confined to a restricted geographic area with individuals of primarily Western European descent, and although broad eligibility criteria are used, results may not be generalizable to other parts of the world.

In conclusion, the spectrum of disease in PLWHIV has changed and non-AIDS comorbidity will be more common as this population continues to age. This will have implications for the clinical care and continue to put new demands on health authorities. This study has the potential to identify novel risk factors for comorbidity in PLWHIV that may be used to guide future interventional studies. Guidelines on screening and monitoring of non-AIDS comorbidity continue to evolve and may help to reduce the burden of non-AIDS comorbidity. Use of data will be confined to the study group, but potential collaborators or request for data can be submitted at cocomo.rigshospitalet@regionh.dk.

## Additional files

Additional file 1: Power calculations. (DOC 53 kb)
Additional file 2: Scan parameters. (DOC 23 kb)

## Abbreviations

ABI: Ankle-brachial index
APRI: AST to platelet ratio index
CACS: Coronary artery calcium score
cART: Combination anti-retroviral therapy
CGPS: Copenhagen general population study
COCOMO: Copenhagen comorbidity in HIV-infection study
COPD: Chronic obstructive pulmonary disease
CT: Computed tomography
CVD: Cardiovascular disease
ECG: Electrocardiography
eNO: Exhaled nitric oxide
HCV: Hepatitis C-virus
HIV: Human immunodeficiency virus
IDU: Illicit drug use
LRD: Liver related disease
MI: Myocardial infarction
NAFLD: Non-alcoholic fatty liver disease
NASH: Non-alcoholic steatohepatitis
PAD: Peripheral arterial disease
PBMC: Peripheral blood mononuclear cells
PCP: *Pneumocystis* pneumonia
PLWHIV: People living with HIV
TE: Transient elastography
