# Supplementary material for: Copenhagen comorbidity in HIV infection (COCOMO) study: a study protocol for a longitudinal, non-interventional assessment of non-AIDS comorbidity in HIV infection in Denmark
Source: BMC Infect Dis. 2016 Nov 26;16:713. doi: 10.1186/s12879-016-2026-9 (PMC5124288; doi:10.1186/s12879-016-2026-9)
Supplement: Additional file 1: — Power calculations. (DOC 53 kb) [file 12879_2016_2026_MOESM1_ESM.doc]

**Additional file 1**

Power calculations were carried out for several endpoints. Figure S1 depicts a power curve for various differences in FEV_1_ declines between people living with HIV (PLWHIV) and uninfected controls. See main document text for further details.

**
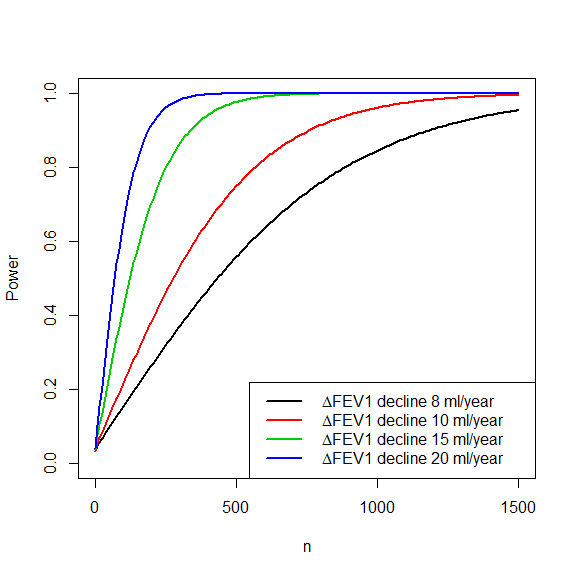
**

**Figure S1.** Power (1-β) is drawn as a function of sample size (n) for various differences in rate of forced expiratory volume in one second (FEV_1_) declines between people living with HIV (PLWHIV) and uninfected individuals. Alpha (α) was taken to be 0.05 and curves are for a balanced design with equal sample sizes in the two groups of interest.

A power-calculation for a cardiovascular disease (CVD) related outcome was based on the probability of detecting a non-calcified coronary plaque on contrast enhanced computed tomography (CT) of the heart of 63% in people living with HIV (PLWHIV) vs. 53% in un-infected controls [1]. A significance level (α) = 0.05, and power (1-β) = 0.9 would require a sample of 489 in each group to detect this difference in a balanced design. A power-calculation for liver related outcomes was carried out based on a probability of having a FIB-4 score ≥ 1.45 (Metavir ≥ F2) at 40% for PLWHIV and 30% for un-infected controls [2]. Assuming α = 0.05, and power = 0.9 would require a sample of 576 in each group in a balanced design. Figures and power calculations were done using G*Power 3.1 and R 3.2.0 [3, 4].
