# Supplementary material for: Copenhagen comorbidity in HIV infection (COCOMO) study: a study protocol for a longitudinal, non-interventional assessment of non-AIDS comorbidity in HIV infection in Denmark
Source: BMC Infect Dis. 2016 Nov 26;16:713. doi: 10.1186/s12879-016-2026-9 (PMC5124288; doi:10.1186/s12879-016-2026-9)
Supplement: Additional file 2: — Scan parameters. (DOC 23 kb) [file 12879_2016_2026_MOESM2_ESM.doc]

**Additional file 2**

The following scan parameters are used: CACS: 120kV (BMI< 28) or 135 kV (BMI>28), automatic exposure control with an SD 55 (min 30 mA and max 300 mA). ECG-triggering with exposure at 75% of the RR-interval is used, and reconstructions are performed with a soft tissue kernel (FC12) and 3/3 mm slice thickness/increment. Unenhanced CT scan of the upper abdomen: A single 16 cm volume scan extending from the left hemidiaphragm and downwards using 40 mA (fixed) and 120 kV. Reconstructions are performed with 1/1 mm and a soft tissue kernel (FC12) using an iterative reconstruction technique (Adaptive Iterative Dose Reduction, AIDR). Visceral adipose tissue scan: A single 8 mm slice at the level of L4 is performed using 120kV and 210 mA. Reconstructions are performed with filtered back projection (FBP) and soft tissue kernel (FC08).Chest CT: A low dose protocol is applied for the full chest examination. Images are acquired at full inspiration using 120 kV and automatic exposure control with an SD of 15. Images are reconstructed with AIDR and both 1/1 mm and 3/3 mm slices. The thin-slice dataset are reconstructed with a lung kernel (FC52) for evaluation of lung parenchyma and airways, and the thicker slices with a soft tissue kernel for evaluation of mediastinum and pleurae. Finally, an additional dataset with 1/1 mm, soft tissue kernel (FC08) and filtered back projection are reconstructed for quantitative measurements of emphysema performed using a dedicated lung density program (Vitrea Vital Images, Minnetonka, MN, U.S.). All participants are screened for contra-indications to beta-blockers and intravenous contrast agent before administration. Prospective ECG-triggering with a target at 75% of the RR-interval is used, using either 100 kV (BMI<28) or 120 kV (BMI>28) and automatic exposure control with SD 40. Reconstructions with filtered back projection, soft tissue kernel (FC03) and 0.5/0.25 mm are transferred to a dedicated workstation with coronary artery evaluation software (Vitrea Vital Images).
